# Book Reviews

**Published:** 1918-01

**Authors:** 


### BOOK REVIEWS

**Books mentioned may be inspected at and ordered through this office. So far as possible, books received in any month will be reviewed in the issue of the second month following. Pamphlets, quarterly and similar periodicals, reports, transactions, etc., will, as a rule, merely be mentioned.**

**Kirke's Handbook of Physiology.** Charles Wilson Greene, Ph. D., University of Mo., Columbia. Wm. Wood & Co., N. Y. 9th American edition, 790 pages, 509 illustrations, many in color. $3.75.

We protest against the price of this book. According to all analogies by number of pages, illustrations, scientific value, etc., it should be $5-$7.50. There is not much to say in reviewing the standard, systematic treatise like this, especially when the book itself has become standard by its wide acceptance through many editions. It is somewhat like being called on to review the Bible or an edition of Shakespeare. In view of the recent interest in examination of aviators, special reference may be made to the illustrations of the semi-circular canals and the discussion of the sense of equlibrium.

---

**Impotence and Sterility,** with Aberrations of the Sexual Function and Sex-Gland Implantation. G. Frank Lydston, M. D., D. C. L. The Riverton Press, 25 E. Washington St., Chicago. 235 pages, illustrated. $4.00.

It is stated that the book is sold only by subscription but we understand that copies may be secured by sending $4.00 to the publishers who will then transmit the book postage prepaid.

Dr. Lydston's work in regard to implantation has aroused so much interest that the profession is, at least familiar with the general subject. This book, however, deals more widely with sexual problems and in a sensible way. We are especially pleased to note that the contention of this journal that legislation should protect the innocence of childhood irrespective of sex, receives the support of Dr. Lydston.

---

**A Manual of Anatomy.** By Henry Erdmann Radasch, M.Sc., M. D., Assistant Professor of Histology and Embryology in Jefferson Medical College. Cloth. Price $3.50 net. Pp. 489, with 329 illustrations. Philadelphia: W. B. Saunders Company, 1917.

This was written for the purpose of filling the need for an anatomy of moderate size for the use of medical students and nurses. One result of the attempt has been so to condense the material that the various anatomic units composing each of the great systems of the body appear to be treated as related structures rather than as independent units whose relation to each other and to the body as a whole is merely incidental—an impression which is apt to be given by the voluminous discussions of the larger textbooks on anatomy. This feature is especially noticeable and helpful in the sections dealing with the anatomy and the histology of the nerve system, in which the gross anatomy and the microscopic anatomy are continued uninterruptedly from segment to another; in addition, the various pathways are given separate and rather full consideration, so that impulses may be traced, in a connected manner, from origin to termination.

---

**An Introduction to the History of Medicine.** With medical chronology, suggestions for study and bibliographic data. By Fielding H. Garrison, A.B., M.D., Principal Assistant Librarian, Surgeon General's office, Washington, D. C. Octavo, 904 pages; illustrated. Second edition. Revised and enlarged. Cloth. Price $6.50 net. Philadelphia and London: W. B. Saunders Company, 1917.

The second edition of Dr. Garrison's book arouses anew the enthusiasm we expressed when reviewing the first edition. As a mere compilation of facts the book alone astonishes by its amazing accuracy. We have read large sections of this book with a keenly critical eye and with the exception of one or two instances, we have not found an error of either commission or of omission. The erudition and research involved in writing this history seems more the result of a lifetime than a matter of a few years. When, in addition, we find our facts so beautifully and logically marshalled, a critique and a sense of historical values there are almost infallible, a delicious characterization and a literary style unequalled among medical men in this country, we can readily perceive why this book has been received with such profound approbation. We repeat that Garrison's book work is one of which American medicine may well be proud.

**The Medical Record Visiting List For 1918.** Handsomely bound in red morocco with flap, pencil, etc. $1.75. William Wood & Co., New York City.

The text portion of the Medical Record Visiting List for 1918 contains a calendar, Estimation of the Probable Duration of Pregnancy, Obstetric Calendar on Folding Chart, Approximate Equivalents of Temperature, Weight, Capacity, Measure, etc., Maximum Adult Doses by the Mouth, in Apothecaries and Decimal Measures, Drops in a Fluid Drachm of different liquids under different conditions, Solutions for Sub cutaneous Injection, Diagnostic Hints on Contagious Diseases, Poisons and their Antidotes, Emergencies, Artificial Respiration, Signs of death, Hints on the Writing of Wills, Table of Signs, Visiting List with special memoranda, Consultation Practice, Obstetric Engagements, Record of Obstetric Practice, Record of Vaccinations, Register of Deaths, Nurses' addresses, Addresses of patients and others, and a cash account.

---

**Physicians' Visiting List for 1918.** Substantially bound in soft black leather, with flap and pencil and pocket. $1.25. P. Blakiston's Sons & Co., Philadelphia, Pa.

The Physician's Visiting List for 1918 contains besides blank pages for memoranda, addresses, engagements, accounts and records of births and deaths, a special division devoted to a calendar, Utero-Gestation table, American table of mortality, Poisoning, Weights and measures, Comparison of thermometers, Table of signs, Asphyxia and Apnoea, Dose table, Isolation periods in infectious diseases and a height and weight table.

